# Electrochromic Adaptive Solar Heater and Sub‐Ambient Passive Daytime Radiative Cooler

**DOI:** 10.1002/adma.74359

**Published:** 2026-07-29

**Authors:** Chenxi Sui, Qizhang Li, Yu Han, Xinyu Dou, Gangbin Yan, Jiadong Liu, Blake Harris, Jinlei Li, Alexander S. Filatov, Qingsong Fan, Ching‐Tai Fu, Po‐Chun Hsu

**Affiliations:** ^1^ Pritzker School of Molecular Engineering University of Chicago Chicago Illinois USA; ^2^ Department of Materials Science and Engineering Stanford University Stanford California USA; ^3^ Department of Earth System Science Stanford University Stanford California USA; ^4^ Department of Chemistry University of Chicago Chicago Illinois USA

**Keywords:** efficient energy use, electrochromism, energy consumption, environmental science, HVAC, materials science, radiative cooling, solar energy, thermal emittance

## Abstract

Heating, ventilation, and air‐conditioning (HVAC) systems are major contributors to global energy consumption, underscoring the urgent need for energy‐efficient building envelope technologies. Synergistic solar and radiative electrochromism offers a promising solution by leveraging the sun and outer space as sustainable thermodynamic resources. In this study, we demonstrate a dual‐mode electrochromic device that enables reversible, non‐volatile switching between solar heating and sub‐ambient radiative cooling, achieving annual energy savings of 73.7 MBtu and CO_2_ emission savings of 4063 kg CO_2_ in specific U.S. climate zones. The device can achieve true sub‐ambient passive daytime radiative cooling by 1°C in the cooling mode and raise it by 33°C above ambient in the heating mode during daytime operation, controlled by electrical voltage. The device employs electrodeposited lossy amorphous Cu‐Bi nanoclusters on defect‐activated monolayer graphene, yielding high optical contrast between cooling (solar absorptance *α* = 7.99%, thermal emittance *ε* = 93.58%) and heating (*α* = 47.7%, *ε* = 20.14%) modes, with outstanding long‐term durability. This work paves the way for scalable, durable, and dynamic thermal regulation, advancing sustainable building technologies.

## Graphical abstract

An electrochromic Cu–Bi nanocluster device dynamically switches between solar heating and daytime sub‐ambient radiative cooling without mechanical motion. Defect‐activated graphene, minimized electrolyte loss, and pulse‐assisted reversible electrodeposition enable opposite solar and mid‐infrared modulation in one opaque platform, establishing a design pathway for adaptive thermal regulation across seasons.

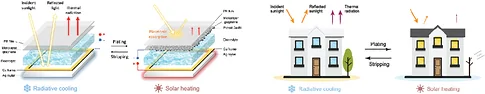

## 1 Introduction

Buildings consume 30% of global energy, with substantial heating and cooling loads required throughout the year [1, 2, 3, 4]. Nearly 17% of the building energy consumption comes from heating, ventilation, and air‐conditioning (HVAC) systems. This energy demand contributes to high operational costs and considerable greenhouse gas emissions, underscoring the urgent need for innovative solutions to improve energy efficiency. A promising approach to addressing this challenge is to leverage the sun (5800 K) and the deep universe (3 K) for passive solar heating and radiative cooling, utilizing these inexhaustible thermodynamic heat/cold resources to regulate building temperatures with minimal energy input [3, 5, 6, 7, 8, 9, 10, 11, 12, 13, 14, 15]. However, due to the seasonal or even diurnal variation in weather conditions, the static and single‐function approach of radiative cooling or solar heating alone cannot address the dynamic thermal demands of buildings effectively [16, 17, 18]. In many places of the world with appreciable seasonal temperature variation, implementing single‐function heating or cooling can incur even higher year‐round energy consumption [16, 19]. The ideal net‐zero‐energy building should dynamically optimize its optical properties in response to changing weather conditions, providing adaptive thermal management that balances heating and cooling requirements in real‐time. Ideally, in hot days, low solar absorptivity combined with high thermal emissivity facilitates radiative cooling. In contrast, in cold days, high solar absorptivity paired with low thermal emissivity enables efficient solar heating [20].

Electrochromic devices are particularly well‐suited for implementing dynamic and on‐demand tuning of building optothermal conditions [21, 22]. They are compatible with existing building electricity systems, have low energy consumption, and allow on‐demand switching between thermal states. Electrochromic devices, which dynamically regulate solar radiation through external voltage stimuli, offer significant potential for reducing building energy consumption and improving thermal comfort. However, traditional electrochromic technologies face key challenges in achieving simultaneous solar and mid‐infrared optical tuning due to the narrow‐band transmission of transparent electrodes [23, 24, 25, 26]. As a result, their tunable range is primarily limited to the solar spectrum, often overlooking the benefits of mid‐infrared modulation [27, 28, 29]. Similarly, mid‐infrared electrochromic devices have been developed but largely missing the modulation of solar heat gain [30, 31, 32, 33, 34]. Although recent advancements have developed ultra‐wideband transparent electrodes capable of addressing this challenge, low optical tuning contrast in the solar spectrum has hindered the ability to achieve sub‐ambient radiative cooling, thus limiting energy‐saving potential [20]. It is worth noting that only the true sub‐ambient radiative cooling (that is, device temperature lower than the terrestrial air temperature that is measured without direct or indirect sunlight) can be considered thermodynamically advantageous compared with the trivial convective or conductive cooling [35].

**FIGURE 1:**
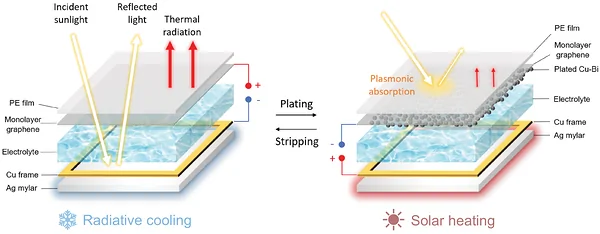
Schematics of the electrochromic adaptive solar heater and radiative cooler.

In this study, we address these challenges by developing an innovative electrochromic material and device system capable of reversible and non‐volatile switching between solar heating and sub‐ambient radiative cooling, catering to the dual thermal management needs of buildings. Through advanced device engineering, temporal modulation of electrochemical driving force, and the integration of a Cu‐Bi amorphous nanocluster on defect‐activated monolayer graphene, we achieve synergistic solar and mid‐infrared optical tuning with a remarkable optical tuning contrast between radiative cooling (solar absorptance, *α* = 7.99%; thermal emittance, *ε* = 93.58%) and solar heating (*α* = 47.7%; *ε* = 20.14%) modes. Optical modeling and material characterization elucidate the underlying mechanisms of the high‐performance optical tuning and electrodeposition process. Comprehensive building energy simulations reveal that this electrochromic adaptive solar heater and radiative cooler can save up to 73.7 MBtu annually in specific U.S. climate zones. This breakthrough provides a scalable and effective route for enhancing energy savings and decarbonizing the building sector (Figure 1).

## 2 Pulse Electrodeposition

Previous studies have demonstrated that ultra‐wideband transparent electrodes, incorporating polyethylene (PE) film, a gold microgrid, and monolayer graphene, can achieve synergistic solar and mid‐infrared tuning [20]. Because of the weak out‐of‐plane chemical interactions, graphene electrodes require a Pt nanoparticle seed layer to enable uniform and reversible metal electrodeposition. Without the Pt layer, metal deposition predominantly occurs around the metal grid, resulting in reduced optical tuning efficiency and reversibility. Unfortunately, this Pt nanoparticle seed layer also strongly absorbs sunlight via localized surface plasmon resonance, preventing the device from achieving a sub‐ambient radiative cooling state [20].

While it is tempting to eliminate both the gold microgrid and the nano‐Pt layer to enhance the device's electrochemical tuning efficiency and optical performance, the removal of these conductive components introduces a critical issue: the intrinsic electrical conductivity of monolayer graphene alone is insufficient compared to the modified transparent electrode [20, 36]. This results in a significant ohmic potential drop across the graphene plane, impairing the uniformity of electrodeposition and reducing optical tuning efficiency. To overcome this limitation, we employed pulse electrodeposition, a technique used in metal plating to control deposition morphology [37, 38, 39]. The large transient voltage overcomes the nucleation threshold overpotential for the entire graphene electrode, creating densely distributed metal nanoparticles that not only increase the electrical conductance but also catalyze the subsequent growth. By leveraging this approach, we improved the uniformity and density of metal deposition, effectively compensating for the absence of the conductive grid and Pt modification. Note that removing optically parasitic conductive grids and Pt seed layers is essential for reducing solar loss and enabling sub‐ambient cooling, but it also makes reversible electrodeposition on ultrathin graphene more challenging due to lateral resistance, nonuniform nucleation, and poorer cycling stability. Therefore, pulse‐assisted Cu–Bi electrodeposition is not used here as a routine plating optimization, but as an enabling electrochemical strategy to restore uniform deposition and reversible switching under the stringent optical‐loss constraints of a sub‐ambient electrochromic radiative cooler. This reflects the coupled optical–electrochemical design principle required for dynamic dual‐mode thermal regulation.

**FIGURE 2:**
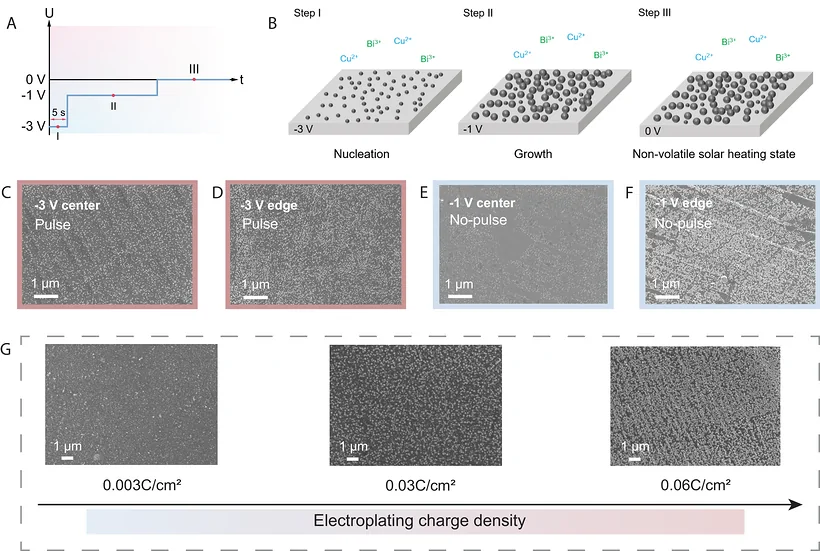
Pulse electrodeposition process. (A) Voltage profile of the pulse electrodeposition process. (B) Schematic representation of material growth during each step. (C–F) Scanning electron microscopy (SEM) images showing metal deposition at different locations on the graphene electrode under varying voltages. (C) and (D) correspond to pulse deposition, and (E) and (F) correspond to no‐pulse deposition. (G) SEM images illustrating metal deposition at different charge densities.

Figure 2A illustrates the voltage profile of the pulse electrodeposition process of Cu‐Bi alloy, which comprises three distinct steps. Step I involves a short‐duration, high‐voltage process designed to uniformly deposit a thin layer of Cu‐Bi nanoparticles across the graphene surface. This phase corresponds to the nucleation step (Figure 2B). Applying a higher deposition voltage (−3 V) overcomes the potential drop caused by monolayer graphene, enabling uniform metal nanoparticle deposition with consistent morphology across both the center and edge of the electrode (Figure 2C,D). In contrast, under conventional deposition voltage (−1 V), most of the metal accumulates near the electrode's edges due to the ohmic drop (Figure 2E,F). In addition to microscale characterization, macroscale optical imaging further validates this phenomenon (Figure S1). Importantly, the duration of high voltage is short enough to prevent noticeable hydrogen evolution. The resulting uniformly distributed metal nanoparticles improve electrodeposition uniformity during subsequent steps. In essence, this step works as an in situ and redox‐reversible modification of graphene with metal nanoparticles to enhance its electrochemical properties, successfully circumventing the undesirable solar absorption from the nano‐Pt modification. Step II changes the potential to −1 V, allowing the deposited metal particles to grow larger and denser (Figure 2B). As the electrodeposited charge density increases, the nanoparticles coalesce into a more continuous layer (Figure 2G), and the voltage is removed after the optical property stops changing. The device is non‐volatile because the optical state is stored as a deposited Cu–Bi solid phase, and in our electrolyte there are no chemical oxidants/etchants that would spontaneously dissolve Cu or Bi at open circuit. Using a Cu^2^^+^/Cu^0^ counter electrode further stabilizes the electrolyte redox environment by acting as a redox reservoir, suppressing uncontrolled composition drift and self‐dissolution pathways. Consequently, no holding power is required—the deposited state is retained at zero bias and can only be removed by applying a reverse potential to intentionally oxidize and strip the metal. To describe the pulsing electrochemistry more clearly, during the deposition step, we applied a two‐stage potential program. Specifically, we first held the working electrode at –3 V for 5 s to initiate rapid nucleation and form a uniform seed layer. We then immediately reduced the potential to –1 V for continued electrodeposition until the accumulated areal charge reached 60 mC cm^−^^2^. Therefore, within each deposition cycle, the potential is switched only once (from –3 V to –1 V). If the –3 V pulsing duration is too short, a sufficiently uniform seed layer cannot be established, and the deposition uniformity deteriorates. Conversely, if the –3 V pulsing duration is too long, mild hydrogen evolution (HER) can occur; the resulting microbubbles can locally block ion transport and active sites, which also compromises uniformity. In our operating window, the particle size is not strongly affected by the –3 V pulse because the pulse duration is brief; instead, the final particle size is governed primarily by the subsequent –1 V growth stage. As a result, longer –1 V deposition (i.e., larger delivered charge during the growth step) leads to larger particle sizes.

## 3 Absorption Enhancement by Amorphous Metal

**FIGURE 3:**
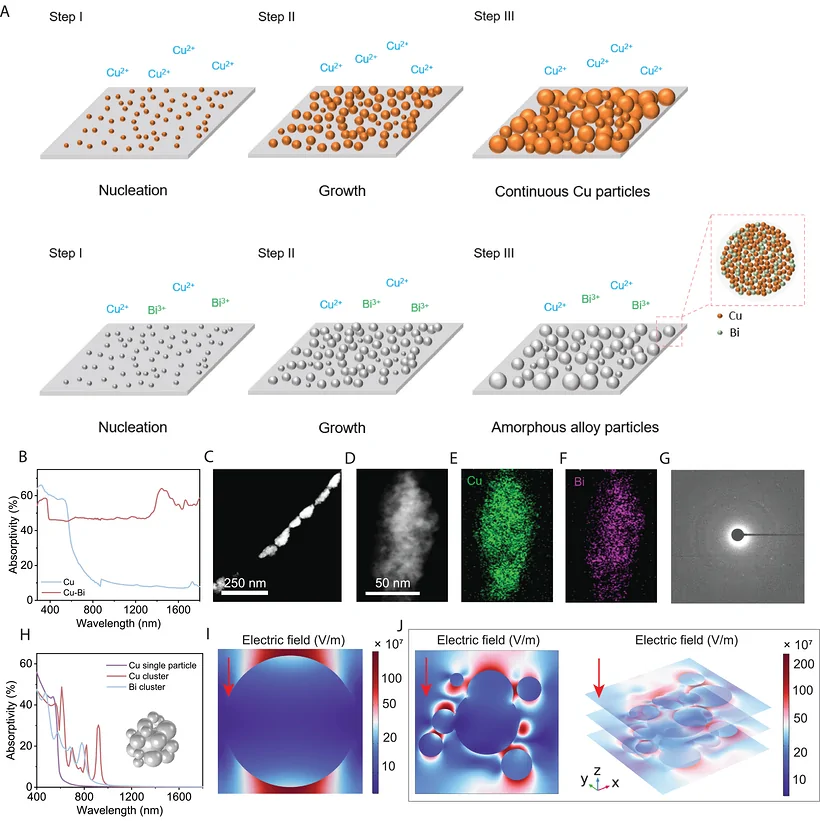
Characterization and optical properties of amorphous Cu‐Bi nanoparticles. (A) Schematic illustration of the electrodeposition process for amorphous Cu‐Bi nanoparticles. (B) Comparison of solar absorptivity between electrochromic devices using Cu and Cu‐Bi electrolytes. (C,D) Cross‐sectional STEM images of the deposited Cu‐Bi nanoparticles. (E–G) EDS mapping and electron diffraction patterns of the Cu‐Bi nanoparticles. (H) Simulated solar absorptivity of various materials with different morphologies, highlighting the optical advantages of the Cu‐Bi nanocluster. (I,J) Simulated electric field distribution around a single Bi nanoparticle and a cluster of Bi nanoparticles at *λ* = 550 nm. Specifically, the first image in (J) shows the cross‐section on the *xy*‐plane at the middle position along the *z*‐axis, while the second image presents a series of cross‐sectional views on various *xy*‐planes. The red arrow specifies the direction of electromagnetic wave propagation.

During the growth stage of electrodeposition, we observed that the solar absorptivity continues to increase until it saturates, an intriguing phenomenon distinct from that of Cu. In Cu electrodeposition, the solar absorptivity tends to drop significantly when Cu nanoparticles begin to coalesce [40]. This is because the merged Cu nanoparticles become larger and larger compared with the skin depth, which exhibits the mirror behavior rather than localized surface plasmon resonance. On the other hand, the thermal emissivity decreases monotonically as the charge density increases. So, what makes Cu‐Bi so special? We speculate that certain microstructural reasons prohibit coalescence from the optical material science standpoint. Further x‐ray diffraction characterization indicates that Cu‐Bi has unusually low crystallinity (Figure S2). Compared to crystalline metals, amorphous metals hold greater potential for sunlight absorption due to their lower intrinsic electrical conductivity [41]. Morphologically, clusters of small nanoparticles can capture more light compared to a single nanoparticle of similar size. It is therefore hypothesized that the immiscibility of Cu and Bi can hinder crystallization and promote the formation of clusters of small nanoparticles (Figure 3A) [42]. By utilizing Cu‐Bi electrolyte, it becomes possible to create amorphous metal films that exhibit both low emissivity and high solar absorptivity.

To validate this, we measured the solar absorptivity of devices fabricated with Cu and Cu‐Bi electrolytes (Figure 3B). The results show that the Cu‐Bi device achieves significantly higher solar absorptivity. For Cu, only the interband transition for wavelengths below 600 nm contributes to the absorption. The morphology and elemental distribution of the deposited metal nanoparticles were characterized using cross‐sectional scanning transmission electron microscopy (STEM). High‐angle annular dark‐field (HAADF) imaging revealed a metal film composed of nanoclusters approximately 50 nm in diameter (Figure 3C). Zooming into a single cluster (Figure 3D–F), energy‐dispersive x‐ray spectroscopy (EDS) mapping confirmed that Cu and Bi are evenly distributed with a Cu:Bi atomic ratio of 3:1. The reduction potentials of Cu and Bi in the same electrolyte environment are similar (theoretical values are both 0.281 V in our electrolyte) and exceed the applied voltage (−1 V), allowing the simultaneous deposition of both metals (Figure S3). Since Cu and Bi are immiscible [43], this single cluster is essentially composed of numerous smaller Cu and Bi nanoparticles. Furthermore, the selected area electron diffraction (SAED) pattern (Figure 3G), which lacks any noticeable crystalline structure, confirms the amorphous nature of the deposited metal. While amorphous/glassy metals are traditionally synthesized through rapid quenching under stringent thermal processing conditions, we demonstrate that the electrodeposition method can also produce amorphous metal films at room temperature, an outcome of rapid solidification from the ionic form.

To further elucidate the enhanced solar heating performance, we used finite element modeling (COMSOL Multiphysics) to simulate the solar absorptivity of Cu and Bi with different morphologies. The refractive indices of Cu and Bi are built into the software library. Two models were analyzed: a single large particle and a cluster of smaller particles with equivalent overall size (Figure 3H, inset). The simulation results show that nanocluster morphology significantly enhances solar absorptivity for both Cu and Bi compared to single Cu particles. Electric field distribution simulations indicate that the clusters exhibit stronger resonances, further enhancing their light‐harvesting capability (Figure 3J). This highlights the critical role of morphology in improving the optical performance of the Cu‐Bi nanoclusters. While previous works have utilized Cu–Bi for color‐neutral electrochromics, the optical mechanism has not been fully explained [28, 44]. Here, our simulation offers a clearer insight into how nanocluster morphology enhances light absorption, thereby complementing and advancing this understanding. More comprehensive experimental and theoretical study further confirmed that the outstanding optical performance is indeed generated from amorphous Cu‐Bi nanocluster nature (Supporting Discussion: S3 and S4).

## 4 Defect‐Activated Graphene for Enhanced Electrodeposition

**FIGURE 4:**
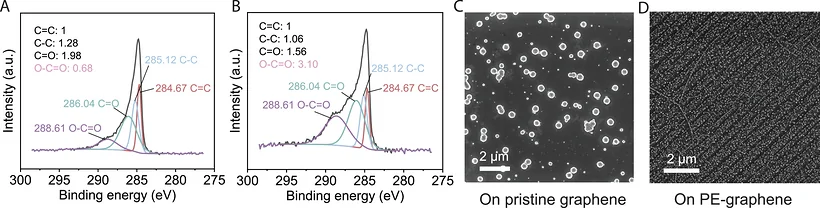
Graphene modification for improving electrodeposition. (A,B) C K‐edge XPS analysis for pristine and transferred graphene. (C,D) SEM images of electrodeposited Cu‐Bi on pristine and transferred graphene.

Pristine monolayer graphene typically exhibits limited electrochemical active sites for the electrodeposited metals, resulting in sparse and non‐uniform electrodeposition. While the pulsed electrodeposition approach resolves the non‐uniformity caused by the ohmic loss, graphene's out‐of‐plane chemical inertness can cause non‐uniformity at the micron scale. To address this, modifying the graphene to introduce additional nucleation sites is crucial for achieving dense and uniform metal deposition. Our findings indicate that the hot‐press transfer process of graphene onto PE film and the oxidative etching of Cu foil inherently introduce O─C═O defects, as confirmed by x‐ray photoelectron spectroscopy (XPS) analysis (Figure 4A,B). These functional groups have strong positive correlation with the uniformity and density of metal deposition compared to pristine graphene (Figure 4C,D).

We attribute this enhancement to the role of O─C═O functional groups as localized nucleation sites. These defect sites disrupt the otherwise chemically inert sp^2^ carbon network, creating localized regions with higher surface energy and charge localization, which in turn can attract metal cations and anchor the electrodeposited metal, promoting more uniform and continuous growth of the metal layer across the graphene surface. An optimal defect (functional group) density is expected for defect‐activated graphene electrodes. Introducing O─C═O functionalities increases nucleation‐site density and promotes uniform initial seeding, but excessive defects disrupt the sp^2^ network, raise sheet resistance, and increase in‐plane potential drop, which can impair electron transport and degrade deposition uniformity and cycling performance. Therefore, the defect density must be engineered within a moderate window that balances nucleation activation and electrical conductivity.

## 5 Device Optical Performance

**FIGURE 5:**
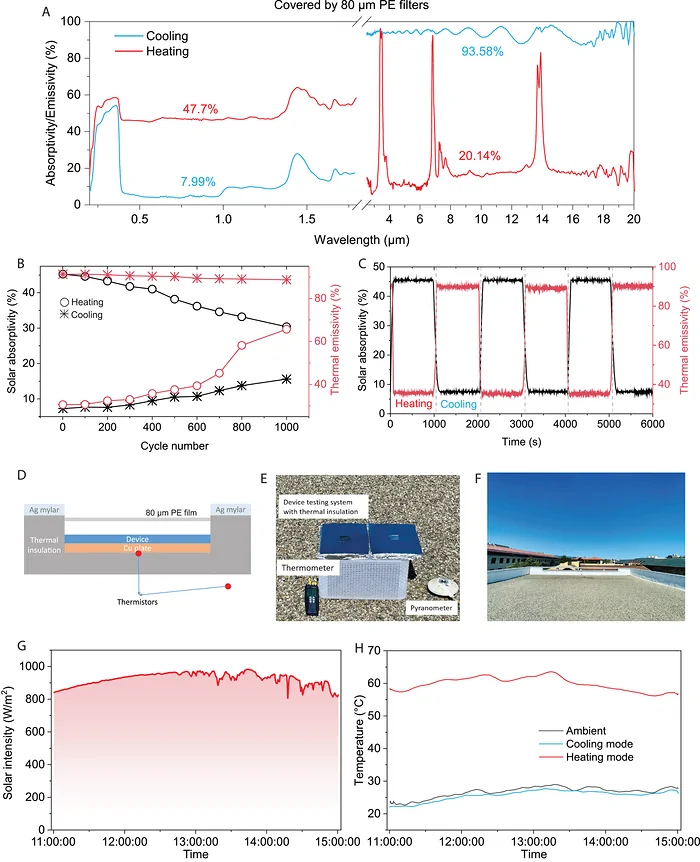
Synergistic electrochemical tuning of solar and mid‐infrared optical properties. (A) UV–vis and Fourier transform infrared spectroscopy (FTIR) measurements illustrating the optical properties of the device in both heating and cooling states. (B) Durability test demonstrating the electrochemical tuning stability over 1000 cycles. (C) Non‐volatile and dynamic tuning of solar absorptivity and thermal emissivity. After the device reaches the heating or cooling state, the applied voltage is turned off, maintaining the state without further electrical input. (D,E) Schematics and photographs of the outdoor test setup. (F) Photograph of the outdoor test location on the rooftop of the Electrical Engineering Building at Stanford University. (G,H) Measured solar intensity and device temperatures under cooling and heating modes.

With the enhanced electrodeposition uniformity achieved by pulsed electrodeposition and defect activation (without the adverse effects of Pt nanoparticles) and the improved solar absorption properties of amorphous Cu‐Bi nanoclusters, the next task is to design the electrochromic device capable of achieving both sub‐ambient radiative cooling and solar heating. In particular, sub‐ambient radiative cooling requires substantial solar reflectance, so any undesired absorption should be further minimized by device engineering. First of all, we optimized the electrolyte thickness to minimize the electrolyte optical path while ensuring enough ion species areal density for electrodeposition (Figure S4). To accomplish sub‐ambient radiative cooling, we further enhanced the device's solar reflectivity by applying a hot‐pressed 80 µm PE film as a top cover filter (Figure S5). Figure 5A illustrates the full spectrum of the device in both heating and cooling states. In the heating state, the device achieves a solar absorptivity of 47.70% and a thermal emissivity of 20.14%. Conversely, in the cooling state, the device exhibits a solar absorptivity of 7.99% and a thermal emissivity of 93.58%. The device behaves as a high‐emissivity solar mirror in the radiative cooling mode and a low‐emissivity solar absorber in the solar heating mode (Figure S6). We also did a systematic study for structure/composition‐optical response correlation to better demonstrate the tunability and mechanism (Supporting Discussion: S4). Durability tests confirm that the solar absorptivity and thermal emissivity can be reversibly tuned over 1000 cycles (Figure 5B). Degradations in the tuning contrast of solar absorptivity and thermal emissivity were observed, which can likely be attributed to side reactions such as hydrogen evolution reaction. This phenomenon is indicated by an increase in the pH of the electrolyte (Figure S7), suggesting proton consumption during cycling. Protons play a crucial role in facilitating efficient electrodeposition and stripping processes, and their depletion may adversely affect device performance [28, 40]. Future electrolyte designs can help mitigate this issue. For instance, water‐in‐salt electrolytes can suppress proton‐related side reactions by reducing free water activity [45, 46]. Ionic‐liquid systems eliminate hydrogen evolution and improve electrode reversibility [47]. Alternatively, using chelating agents or operating in mildly alkaline complexing electrolytes can stabilize metal ions and suppress proton reduction [48]. Furthermore, thanks to the non‐volatile nature of the electrodeposition process, the device's heating and cooling states can be dynamically tuned and then maintained without any electricity input, as shown in Figure 5C. Given its exceptional optical tuning capability, we conducted an outdoor test to demonstrate the temperature management performance of the electrochromic device (Figure 5D–F). The test took place on May 14, 2025, on the rooftop of the Electrical Engineering Building at Stanford University. The results show that, under direct sunlight, the device in cooling mode was able to reduce the temperature to approximately 1°C below ambient, while in heating mode, it increased the temperature by an average of about 33°C above ambient (Figure 5G,H). We also systematically evaluated the device stability under multiple real‐world stressors to demonstrate the durability of the device (Supporting Discussion: S5).

## 6 Building Energy and CO_2_ Emission Saving Capability

**FIGURE 6:**
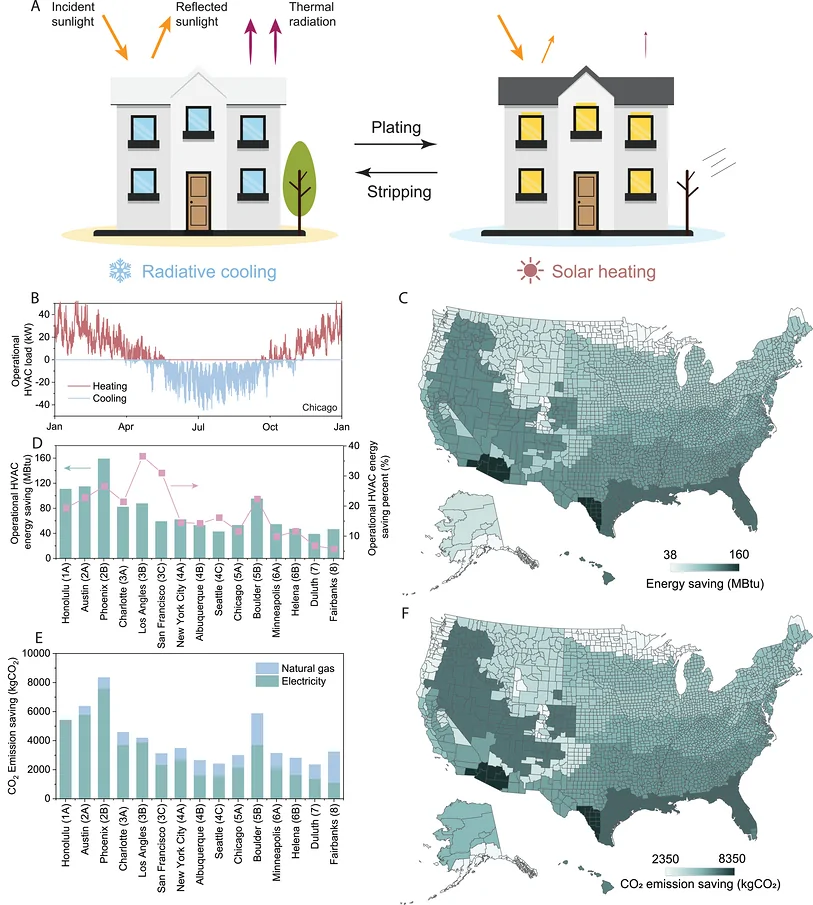
Energy saving capability. (A) Illustration of energy‐efficient buildings equipped with the developed electrochromic device, demonstrating its dual‐mode functionality for heating and cooling. (B) Annual operational HVAC loads for a mid‐rise apartment in Chicago. (C) U.S. energy‐saving map highlighting the regional effectiveness of the device in reducing HVAC energy consumption. (D) Operational HVAC energy savings and corresponding percentage reductions achieved in selected U.S. cities. (E) Annual CO_2_ emission saving in selected U.S. cities. (F) U.S. annual CO_2_ emission saving map.

The exceptional performance of the electrochromic adaptive solar heater and radiative cooler demonstrates its significant potential for reducing HVAC energy consumption in buildings. When installed on rooftops, the device facilitates heat radiation into deep space during hot seasons and absorbs sunlight for heating in cold seasons (Figure 6A). To assess its energy‐saving potential, *EnergyPlus* simulations were conducted for a building equipped with an electrochromic device. Figure 6B illustrates the annual HVAC loads for a mid‐rise apartment building in Chicago (Figure S8), showing pronounced peak demand in winter and summer, along with moderate, rather than negligible, demand during spring and autumn. Following the installation of the electrochromic device, an average energy savings of 73.69 MBtu annually was observed across the U.S. regions. Due to the device's effective sub‐ambient radiative cooling performance, more significant energy savings are realized in warmer climates compared to colder regions (Figure 6C,D). This underscores the need for further advancements in materials and electrochemical optimization to enhance the solar heating efficiency of electrodeposited metals. To highlight the environmental benefits of this electrochromic device, we further calculated the annual CO_2_ emission savings across the U.S. (Figure 6E,F). The trend closely follows the HVAC energy savings, with greater reductions in warmer regions due to the enhanced radiative cooling performance. Additionally, the dynamic electrochromism further reduces CO_2_ emissions associated with electricity consumption, as most cooling demand is met by electricity, whereas heating primarily relies on natural gas. On average, this electrochromic device can help U.S. buildings reduce CO_2_ emissions by approximately 4063 kg CO_2_ per year.

## 7 Discussion

In this study, we successfully developed an electrochromic device capable of dynamically regulating solar radiation and thermal emissivity, providing a promising solution for reducing energy consumption in buildings. The device combines the synergistic tuning of solar and mid‐infrared optical properties, achieving efficient solar heating during cold weather and sub‐ambient radiative cooling during hot weather. By leveraging Cu‐Bi amorphous nanoparticles and optimizing the electrochemical deposition process, we demonstrated the feasibility of this device for year‐round energy savings. The integration of the electrochromic device into building envelopes holds significant potential for reducing HVAC loads and operational energy consumption, especially in regions with hot climates.

The *EnergyPlus* simulations revealed that the electrochromic device could save an average of 73.69 MBtu annually across different regions in the U.S., with more pronounced savings in warmer climates due to its effective radiative cooling capabilities. This suggests that, while the device performs well across various geographic locations, future efforts should focus on enhancing solar heating efficiency, particularly in cooler regions. Challenges remain in optimizing solar heating performance and achieving greater efficiency in colder climates. Nevertheless, the situation may be subject to change given the rising global temperature. While the device's ability to switch between heating and cooling modes based on external conditions offers great promise, further improvements in material selection, electrochemical processes, and device architecture are necessary to fully realize its potential. Additionally, scaling the technology for widespread commercial application will require further research into cost‐effectiveness, durability, and long‐term performance under real‐world conditions.

In conclusion, the electrochromic adaptive solar heater and radiative cooler presented in this work provide a novel and effective approach to building energy management. Its versatility in tuning optical properties in response to ambient conditions offers a sustainable, low‐energy solution for enhancing building energy efficiency. In an electrochemically switchable radiative cooler, reaching the sub‐ambient regime is particularly challenging because transparent electrodes, electrolytes, current collectors, active deposits, and encapsulation layers can all introduce parasitic solar absorption, scattering, thermal leakage, or electrochemical nonuniformity. Therefore, the monolayer graphene electrode, minimized electrolyte thickness, removal of optically lossy metal grids/Pt seeds, and pulse‐assisted Cu–Bi electrodeposition were not isolated optimizations, but necessary design choices to suppress loss pathways while preserving reversible electrochemical switching. This integrated optical–electrochemical design enables the device to cross the stringent sub‐ambient cooling threshold in a dynamic electrochromic architecture. The present outdoor measurement should be viewed as a proof‐of‐concept demonstration of electrochemically switchable daytime sub‐ambient radiative cooling, rather than a complete deployment‐level reliability study across multiple climates and seasons. Although the current cooling temperature drop and heating‐state solar absorptance can be further improved, crossing the sub‐ambient threshold in a dynamic electrochromic device is already a stringent milestone because both low solar heat gain and reversible electrochemical switching must be maintained simultaneously. This result demonstrates a previously unrealized non‐mechanical electrochemical platform that can reversibly switch between solar heating and true daytime sub‐ambient radiative cooling. Continued optimization and research are crucial for addressing the challenges posed by seasonal climate variations and unlocking the full potential of this technology for widespread adoption in energy‐efficient buildings. We also want to mention that this current‐generation graphene transparent electrode still has certain limitations—most notably, when scaled to larger areas it can suffer from a significant in‐plane potential drop. We therefore discuss several feasible strategies to address this scale‐up challenge in the Supporting Discussion S1. Moreover, its non‐destructive retrofitting capability presents an exciting opportunity for enhancing energy performance in existing infrastructure, making it a compelling option for the global energy transition.

## 8 Method

### 8.1 Electrolyte Preparation

The aqueous electrolyte consists of 10 mM Cu(ClO_4_)_2_, 10 mM BiOClO_4_, 1 M LiClO_4_, 10 mM HClO_4_, and 10 wt% poly(vinyl alcohol) for uniform electrodeposition. The whole solution was mixed under 80°C.

### 8.2 Electrochromic Building Envelope Fabrication

The monolayer graphene is purchased from CheapTube Inc., which is CVD‐grown on Cu foil. The Cu‐graphene sample is hot‐pressed onto 20 µm PE film at 99°C, and then the Cu is dissolved in 1 M FeCl_3_ solution. Finally, the sandwiched sample is rinsed with DI water to form a transparent conductive electrode.

The copper tape is used as a counter electrode and is taped on the Silver Mylar. The Kapton tape with 20 µm thickness is used as the separator. Then the transparent electrode is attached to the top to seal the electrode in between.

### 8.3 Cross‐Sectional TEM Characterization

After deposition, the film was embedded in Poly/Bed 812 resin to get cross‐section view images and cut into 90‐nm‐thick slides using an ultramicrotome (Ultracut E, Reichert‐Jung). High‐angle annular dark‐field (HAADF) imaging and selected area electron diffraction (SAED) were conducted with the aberration‐corrected scanning transmission electron microscope JEOL ARM200CF (200 kV) at the University of Illinois at Chicago. EDS spectrum imaging was performed using an Oxford X‐Max 100TLE windowless SDD detector.

### 8.4 Outdoor Temperature Testing

The device was first electrically biased prior to testing to achieve its optimal cooling and heating states. Owing to its non‐volatile nature, the device retained its optical properties throughout the entire testing period. It was then mounted onto a copper plate, which was attached to a vacuum insulation panel to minimize parasitic heat losses. The insulation panel was wrapped in Ag‐coated Mylar for enhanced thermal isolation. Finally, the top surface of the device was covered with 80 µm PE film to partially block solar radiation, thereby enhancing radiative cooling performance and reducing convective heat loss.

### 8.5 Building Energy Saving Calculation

We employed *EnergyPlus*, an open‐source whole‐building energy simulation tool (https://energyplus.net/), to estimate HVAC energy consumption throughout the year. *EnergyPlus* simulates realistic buildings using a heat balance‐based approach to account for all heat transfer processes on both internal and external surfaces. The software incorporates various loads, including equipment, occupants, and lighting, while ensuring occupants’ thermal comfort. It solves the energy balance equation hourly over the entire year, considering the interaction between indoor (room temperature) and outdoor (ambient temperature based on weather data) conditions.

HVAC energy consumption was modeled across different U.S. regions using *EnergyPlus* version 9.4. Fifteen cities were selected to represent the diverse climate zones in the United States. The simulations used hourly weather data from a typical meteorological year (TMY2). The model utilized was the “new‐2004 midrise apartment” from the U.S. Department of Energy. This building had a rectangular design with a floor area of 783.75 m^2^ (length: 46.33 m, width: 16.91 m, height: 12.19 m), four stories, and a window‐to‐wall ratio of 15%. Baseline simulations were conducted with standard wall properties (default settings as downloaded). Cooling and heating loads were calculated on an hourly basis for the entire year.

To model the impact of the electrochromic mid‐IR device at the building level, we modified the radiative property (mid‐IR emissivity) of the out‐layer surface material in *EnergyPlus* according to the measured device states, while the baseline building used the default (normal) building model with built‐in optical properties. For each city, *EnergyPlus* was run to obtain the hourly cooling and heating loads under each emissivity setting. We denote *P*_cool,h_ and *P*_heat,h_ as the hourly cooling and heating loads under the heating‐mode emissivity, and *P*_cool,c_ and *P*_heat,c_ as the hourly cooling and heating loads under the cooling‐mode emissivity. The HVAC energy consumption per hour was then computed as $E=3600\times \max(P_{\mathrm{cool}},P_{\mathrm{heat}})$, where *P*_cool_ and *P*_heat_ are the hourly cooling and heating loads (in W), and the factor 3600 converts power to energy per hour (J). Accordingly, the annual energy consumption for the heating‐mode and cooling‐mode buildings can be calculated as *E_h_* and *E_c_* by summing the corresponding hourly energies over the year. Considering the dynamic tuning capability of the electrochromic device, we further assume that the building can switch between these two emissivity states to minimize HVAC energy demand; therefore, for each hour the building adopts the lower energy cost between the two modes that is $E_{d}=\;\min\left(E_{h},E_{c}\right)$, and the total annual energy cost is obtained by summing up the hourly values of *E*_d_ throughout the year.

### 8.6 CO_2_ Emission Saving Calculation

We calculated the daily CO_2_ emission factor of electricity generation in the US for the latest available year, 2024. The approach follows IPCC (2006) guidelines to ensure consistency and accuracy [49].

The calculation of CO_2_ emission factors relies on multiple datasets:

- Electricity generation data by energy source: Obtained from the U.S. Energy Information Administration's (EIA) Hourly Electric Grid Monitor (https://www.eia.gov/beta/electricity/gridmonitor/), covering various energy sources.
- Emission factors by energy source: Extracted from the U.S. EIA State Electricity Profiles (https://www.eia.gov/electricity/state/unitedstates/state_tables.php), measured in pounds per kilowatt‐hour (pounds/kWh).

The study includes the following primary energy sources: (1) Coal, (2) natural gas, (3) petroleum, and (4) all other energy sources (including fossil fuels, specific geothermal power plants, and other sources).

Electricity generation from hydro, solar, and wind is treated as carbon neutral, in accordance with EIA guidelines.

The CO_2_ emission factor is calculated as follows:

$$\mathrm{EF}\;=\frac{\sum (AD_{k}\times EF_{k})}{\sum AD_{k}}$$

where *k* reflects the energy sources considered in the calculation. In our study, *k* covers three primary fossil fuels types which are coal, petroleum, natural‐gas along with a category labeled "All Other Energy Sources." AD_*k*_ represents the activity data, which refers to the amount of electricity generated by each energy source *k*. The electricity generation data for various energy sources is obtained from the U.S. Energy Information Administration's (EIA) Hourly Electric Grid Monitor. EF_*k*_ represents the emission factor, defined as the amount of CO_2_ emissions produced per unit of electricity generation for energy source *k*. The emission factors for various energy sources are collected from U.S. EIA State Electricity Profiles, as shown in Table S1. Finally, Figure S10 presents the estimated daily CO_2_ emission factor of electricity generation in the U.S. for 2024, measured in kg CO_2_ per kWh. The emission factor of natural gas for homes is collected from the U.S. Energy Information Administration's (EIA) Carbon Dioxide Emissions Coefficients by Fuel (https://www.eia.gov/environment/emissions/co2_vol_mass.php). The CO_2_ factor of natural gas for homes and businesses is 52.91 kg CO_2_ per million Btu.

## Author Contributions

P.‐C.H. and C.S. conceived the idea. C.S. performed experiments, building energy simulation, and corresponding data analyses. Q.L. conducted experiments, COMSOL optical simulation, and data analyses. Y.H. conducted the cross‐sectional TEM and EDX mapping. X.D. and C.S. did the CO_2_ emission calculation. C.S., Q.L., and P.‐C.H. wrote the manuscript with input from all co‐authors.

## Conflicts of Interest

P.‐C.H. and C.S. have a 2021 US patent application (63/256,136). The other authors declare no conflicts of interest.

## Supporting information




**Supporting File**: adma74359‐sup‐0001‐SuppMat.docx.

## Data Availability

All data needed to support the conclusions in the paper are present in the manuscript and/or Supporting Information. Additional data related to this paper may be requested from the corresponding authors.
