# Supplementary material for: Electrochromic Adaptive Solar Heater and Sub‐Ambient Passive Daytime Radiative Cooler
Source: Adv Mater. 2026 Jul 29;38(53):e74359. doi: 10.1002/adma.74359 (PMC13591661; doi:10.1002/adma.74359)
Supplement: Supplementary file 1 [file ADMA-38-e74359-s001.docx]

Supplementary Information for

**Electrochromic adaptive solar heater and sub-ambient passive daytime radiative cooler**

Sui et al

**Contents Summary:**

Supplementary Figures 1-34

Supplementary Tables 1-3

Supplementary Figure 1. Electrodeposited Cu-Bi on a graphene transparent electrode with different potentials, -1 V and -3 V.

Supplementary Figure 2. XRD 2θ scan of electrodeposited Cu and Cu-Bi on graphene. Cu shows clear crystallinity peaks but Cu-Bi does not show any noticeable crystallinity.

Supplementary Figure 3. Linear sweep voltammetry of the single metal salt electrolyte with only Cu and Bi. The reduction peak of Cu deposition is close to the Bi deposition. The concentration of Cu and Bi sources are 10 mM, and the Ag/AgCl reference electrode is soaked in 3 M KCl.

Supplementary Figure 4. Thickness optimization of the aqueous electrolyte layer. After removing the Pt modification layer, the final challenge is to achieve subambient radiative cooling. In the device structure, the aqueous electrolyte is the primary contributor to solar absorption, as water is a strong near-IR absorber that significantly enhances solar heating. Therefore, to enable subambient radiative cooling, it is essential to reduce the solar absorption of the electrolyte. We calculated the solar absorption of water as a function of its thickness and found that minimizing the electrolyte thickness is critical. To address this, we utilized a 20 µm (labeled by red arrow in the right data plot) thick Kapton tape as a spacer between the transparent electrode and the silver mylar, effectively reducing the electrolyte thickness to its optimal limit.

Supplementary Figure 5. Transmittance of PE film with different thickness.

Supplementary Figure 6. Optical image of the electrochromic device in heating and cooling mode.

Supplementary Figure 7. pH value of electrolyte after 1000 cycles.

Supplementary Figure 8. *EnergyPlus* mid-rise apartment model.

Supplementary Figure 9. Schematics of ultra-microtome sample fabrication.

Supplementary Figure 10. Daily CO_2_ emission factor of electricity generation in the U.S. (2024) (*unit: kg CO_2_ per kWh*).

| Energy Source | Emission Factor (pounds CO_2_ per kWh) |
| --- | --- |
| Coal | 2.31 |
| Natural gas | 0.96 |
| Petroleum | 2.46 |
| All other energy sources | 0.81 |

Supplementary Table 1. Emission factors by energy source in electricity generation in the U.S. *All other energy sources* include fossil fuels, some types of geothermal power plants, and other sources. Petroleum includes petroleum liquids (mainly distillates and residual fuel oil) and petroleum coke. Data are for utility-scale electric power plants, including combined heat and power plants.

**Supplementary Discussion 1**

While the present monolayer-graphene transparent electrode enables low-parasitic optical operation and uniform switching at the demonstrated device size, scaling the device to substantially larger lateral dimensions will introduce additional constraints that must be addressed. The most critical limitation is the increasing in-plane ohmic loss of the transparent electrode, which can generate a non-negligible spatial potential gradient across the active area. As the device area increases, the lateral distance over which electrons must be transported to sustain the electrodeposition/stripping current becomes longer, and the resulting potential drop (set by the electrode sheet resistance and the local current distribution) can lead to location-dependent electrochemical driving forces. In practice, this manifests as non-uniform nucleation and growth kinetics between regions close to the electrical contact and regions farther away, potentially producing gradients in deposit coverage, morphology, and optical state, and ultimately compromising switching contrast and cycling stability at large scale. In addition, transient high-bias nucleation steps, while beneficial for initiating dense and spatially uniform seeding in small-area devices, may become more difficult to apply uniformly in large-area geometries because local overpotential variations can also influence side reactions (e.g., mild hydrogen evolution) and local mass transport, further amplifying nonuniformity.

Several engineering strategies can be envisioned to mitigate the scale-up-induced potential drop while retaining the device’s low-loss optical design philosophy. A direct approach is to reduce the effective lateral resistance by integrating an ultrafine conductive metal grid (e.g., Au or Al) with micro-/nano-fabricated linewidth and pitch on or beneath the graphene electrode. Such a grid effectively shortens the electron-transport length scale and distributes current more evenly across the device, suppressing potential gradients that drive nonuniform electrodeposition. Importantly, when the grid features are sufficiently narrow and sparse, the added optical parasitic absorption and reflection can be minimized; however, a residual tradeoff is unavoidable, as any metallic current collector can introduce some optical loss, particularly in the solar band, and thus may reduce the achievable modulation contrast. Another scalable route is to increase the electrode conductivity by forming a thicker, more conductive graphene-based network, for example via spray-coating graphene nanoplatelets (or related conductive inks) onto the graphene electrode to create additional percolation pathways. This strategy can substantially lower sheet resistance and relax the scale-up constraint, but it likewise introduces a conductivity–transparency tradeoff: additional graphene layers and junctions can increase broadband optical absorption/scattering and therefore may reduce the optical switching contrast, especially for designs that require ultra-low parasitic loss to reach sub-ambient daytime cooling. In future iterations, these strategies can be combined with optimized contact design (e.g., multi-side busbars, distributed contact points) and cell geometry engineering to further homogenize current distribution. Overall, we anticipate that large-area deployment will require deliberate co-optimization of electrical uniformity (minimizing in-plane potential drop) and optical parasitic loss (preserving wideband transparency), and the appropriate balance will depend on the target application, device size, and acceptable tradeoffs in switching contrast and sub-ambient cooling performance.

**Supplementary Discussion 2**

“To clarify the distinction between the present work and previously reported electrochromic thermal regulation devices, we summarized representative state-of-the-art systems in Supplementary Tables 2 and 3. Although several electrochromic or electrically tunable devices have demonstrated impressive optical modulation, most existing systems are optimized for only one spectral regime or rely on additional architectural degrees of freedom. For example, several devices primarily regulate the solar band by changing transmittance, reflectance, or visible/NIR color, while their mid-IR emissivity remains essentially fixed. Other systems focus mainly on mid-IR emissivity modulation for thermal camouflage or radiative insulation, but do not dynamically switch the solar absorption/reflection needed for active heating–cooling regulation. Some recently reported dual-band systems achieve large optical contrast, but require mechanical flipping, stacked/multilayer architectures, or separate optical components to realize the full heating/cooling functionality. While these approaches are effective, they are fundamentally different from a monolithic, non-mechanical electrochromic device, in which both solar and mid-IR properties are dynamically and reversibly regulated within a single integrated platform.

The central distinction of this work is that the device simultaneously realizes opposite-trend dual-band modulation in an opaque electrochemical architecture: the heating state requires high solar absorption and low mid-IR emissivity, whereas the cooling state requires low solar absorption and high mid-IR emissivity. Achieving these two spectrally opposite states in one electrochromic device is substantially more challenging—and more critical—than single-band tuning because the electrode, electrolyte, current collector, and active material must all satisfy broadband optical and electrochemical constraints at the same time. In particular, true daytime sub-ambient radiative cooling imposes a stringent thermodynamic requirement: parasitic solar absorption and optical losses from the electrolyte, electrodes, and seed/current-collector structures must be sufficiently suppressed so that the device can maintain a net outward radiative heat flux even below ambient temperature. Therefore, the monolayer graphene transparent electrode, minimized electrolyte thickness, removal of optically lossy Pt seed/grid structures, and pulse-assisted Cu–Bi electrodeposition are not merely isolated optimizations, but necessary elements of an integrated electrochemical–optical design strategy.

Compared with devices that report larger optical contrast in a single band or larger apparent temperature modulation across different device architectures, the present device demonstrated, for the first time, an integrated function: electrically switchable, non-mechanical, opaque, dual-band thermal regulation, with demonstrated true daytime sub-ambient radiative cooling and decent solar heating. This comparison highlights that the key contribution of the present work is not simply maximizing a single performance metric, but establishing a generalizable design pathway for electrochemical dynamic optical devices to cross the demanding threshold from above-ambient thermal modulation to true sub-ambient radiative cooling while retaining reversible solar-heating capability.

It should also be mentioned that, in this work, the outdoor thermal measurement is intended as a proof-of-concept demonstration of electrochromically switchable daytime sub-ambient radiative cooling, rather than a complete deployment-level reliability study across different climates, seasons, and long-term weathering conditions. Although the measured sub-ambient temperature drop is modest, crossing the sub-ambient threshold in a dynamic electrochemical device is itself a stringent milestone, because the device must simultaneously suppress parasitic solar absorption and maintain high mid-IR emission while retaining reversible electrochemical switching. Therefore, the present result should be viewed as an initial demonstration that a non-mechanical electrochromic device can enter the true sub-ambient radiative-cooling regime, with further optical and durability optimization expected in future device generations.

Supplementary Table 2. Summary of state-of-the-art progress in electrochromic radiative cooling.

Supplementary Table 3. side-by-side comparison of state-of-the-art progress in electrochromic radiative cooling. (NR means not reported)

**Supplementary Discussion 3**

We would like to clarify that in our system, the amorphous nanocluster nature of the Cu–Bi deposition is specifically responsible for the enhanced broadband solar absorption, whereas the suppressed long-wave infrared (IR) emissivity is a result of the percolation of the metallic particles. In this framework, the suppressed infrared emissivity can be expected from any electrodeposited metal film, while the broadband solar absorption observed is an unusual phenomenon intrinsic to the amorphous Cu–Bi nanocluster. To rigorously distinguish our findings from generic effects like scattering or thickness, we have conducted additional systematical experiments, as detailed below.

We first measured the complex permittivity ($\epsilon=\epsilon_{1}+i\epsilon_{2}$) of different electrodeposited metals: Cu, Bi, and Cu–Bi, in the visible light wavelength range (380–790 nm). As shown in Supplementary Figure 11, Bi and Cu show typical metal features (negative real permittivity $\epsilon_{1}<0$) while Bi shows higher loss (represented by $\epsilon_{2}/\left| \epsilon_{1} \right|$) than Cu. One might naturally expect the Cu–Bi alloy to exhibit an intermediate metallic response. However, counterintuitively, the deposited Cu–Bi exhibits a positive real permittivity ($\epsilon_{1}>0$) and a significantly high imaginary part ($\epsilon_{2}\sim\left| \epsilon_{1} \right|$) across the entire visible spectrum. The positive $\epsilon_{1}$ observed in Cu–Bi cannot be accounted for by effective medium theory, as both constituent metals (Cu and Bi) exhibit negative $\epsilon_{1}$ individually, and effective medium models would generally yield a negative $\epsilon_{1}$ regardless of composition ratio. This rules out a geometric mixing origin and points to an intrinsic modification of the electronic response upon amorphous co-deposition.

Supplementary Figure 11. Complex permittivity ($\epsilon=\epsilon_{1}+i\epsilon_{2}$) of electrodeposited metals measured by variable-angle spectroscopic ellipsometry. All metals were deposited on silicon wafers coated with a 150 nm Au layer with the same deposition charge density of 400 mC/cm². Optical constants were extracted using a B-Spline model with Kramers–Kronig consistency enforced, ensuring the physical validity of the derived n and k values.

Furthermore, we measured the absorptivity of the deposited metals, as shown in Supplementary Figure 12. The results show that Cu–Bi co-deposition yields higher solar absorptivity than either single-metal deposition. This further validates that the observed optical response cannot be explained by a simple mixture of the constituent metals.

Supplementary Figure 12. Measured absorptivity spectra of electrodeposited Cu, Bi, and Cu–Bi films (on Au-coated wafer) collected by UV-Vis-NIR spectrophotometry.

To explore the unusual optical behavior of the deposited Cu–Bi, we conducted infrared spectroscopic ellipsometry to characterize its complex permittivity in the long-wavelength regime (Supplementary Figure 13). Our results reveal that while the Cu–Bi film maintains metallic characteristics in the IR ($\epsilon_{1}<0$), it is exceptionally lossy, as evidenced by a significantly large imaginary part ($\epsilon_{2}$). To gain further insight into the underlying mechanism, we fitted the measured permittivity using the Drude model:

,

where $\epsilon_{\infty}$ is the high-frequency dielectric constant accounting for bound electron contributions, $\omega_{p}$ is the plasma frequency of free carriers, and $\gamma$ is the damping rate describing free electron collisions. Although the microscopic structure of the Cu–Bi film is highly disordered, we consider Drude model remains a reasonable description since the infrared optical response is still dominated by free-carrier dynamics. As a result, the fitted results show excellent agreement with the experimental data (Supplementary Figure 14). The extracted plasma frequency $\omega_{p}$for Cu–Bi (6.53 eV) is lower than that of pure Cu (10.83 eV, from *Appl. Opt. 37, 5271-5283*, *1998*) but remains within the same order of magnitude. However, Cu–Bi exhibits a significantly larger damping rate ($\gamma=2.87$ eV) than Cu (0.03 eV) by roughly two orders of magnitude. Such extreme damping is consistent with the highly disordered amorphous nanocluster structure, where severe structural disorder significantly reduces the electron mean free path, fundamentally driving the material into an overdamped metallic state.

Supplementary Figure 13. Complex permittivity ($\epsilon=\epsilon_{1}+i\epsilon_{2}$) of electrodeposited Cu–Bi measured by variable-angle spectroscopic ellipsometry. The sample is the same as that used in Supplementary Figure 11. The measurement was done with IR-VASE Mark II from JA Woollam Co.

Supplementary Figure 14. Measured and fitted complex permittivity of the Cu–Bi film in the mid-infrared spectral range (4–20 μm). The fitted Drude parameters are: $\epsilon_{\infty}=2.77$, $\omega_{p}=6.53$ eV, and $\gamma=2.87$ eV.

To examine whether the overdamped Drude model can account for the anomalous optical response of the Cu–Bi alloy in the visible range, we first extrapolated the permittivity using the Drude parameters ($\epsilon_{\infty}$, $\omega_{p}$, and $\gamma$) obtained from the IR fitting. As shown in Supplementary Figure 15, although the extrapolated dielectric function does not quantitatively reproduce the measured permittivity across the full visible spectrum, it correctly predicts the onset of positive $\epsilon_{1}$ near 450 nm, which captures the qualitative trend despite the model's inherent limitations in this regime. We further performed a direct Drude fit to the visible-range permittivity data. The optimized parameters yield$\omega_{p}=6.14$ eV and $\gamma=2.87$ eV, in close agreement with those from the infrared fit ($\omega_{p}=6.53$ eV and $\gamma=2.87$ eV), confirming that the free-carrier properties are consistent across spectral ranges. The somewhat larger $\epsilon_{\infty}$ obtained in the visible fit (4.71 vs. 2.77) is expected, as interband transitions near the visible range contribute additional background polarization that is absent in the IR range. The inability to achieve a perfect fit in the visible regime is likewise anticipated: the Drude model captures only free-carrier dynamics and does not account for the strong interband transitions characteristic of Cu- and Bi-based metals in this spectral range.

Nevertheless, the Drude analysis provides meaningful physical insight. According to the Drude model, $\epsilon_{1}=0$ occurs at

.

For conventional metals such as Cu and Au, $\gamma\ll\omega_{p}$, so this zero-crossing falls in the ultraviolet, leaving $\epsilon_{1}<0$ throughout the solar spectrum and conferring strong reflectivity. In the heavily damped Cu–Bi alloy, however, $\gamma$ is comparable to $\omega_{p}$, which red-shifts the zero-crossing into or even beyond the visible range, giving rise to near-zero or positive $\varepsilon_{1}$. While this free-carrier picture does not fully capture the solar-spectrum behavior of Cu–Bi, which also involves interband transitions and nanoscale plasmonic resonances, it establishes a physically grounded framework for understanding the alloy's anomalous optical response and its implications for enhanced solar absorption.

Supplementary Figure 15**.** Drude model fitting of the dielectric function for electrodeposited Cu–Bi thin film in the visible range. Black circles: ellipsometry measurement data; blue dashed line: Drude model using parameters extrapolated from infrared fitting; red solid line: optimized Drude fit to the visible-range data. The fitted Drude parameters are: $\epsilon_{\infty}=4.71$, $\omega_{p}=6.14$ eV, and $\gamma=2.87$ eV.

It is noteworthy that despite the exceptionally high damping rate, the infrared reflectance of the Cu–Bi film remains largely preserved. This is because, in the long-wavelength regime, both the real and imaginary parts of the refractive index (*n* and *k*) remain sufficiently large (e.g., *n* = 7.7 and *k* = 7.8 at 10 µm), resulting in a substantial impedance mismatch between the lossy metal and air. This mismatch effectively suppresses light penetration and sustains high surface reflection. To verify this, we calculated the theoretical IR reflectance using the experimentally measured complex permittivity. As shown in Supplementary Figure 16, the calculated reflectance is approximately 80% in the long-wave IR range, which is in excellent agreement with our experimental measurements. These results confirm that the amorphous nanocluster structure can simultaneously support strong broadband solar absorption and maintain high IR reflectance for efficient thermal management.

Supplementary Figure 16**.** Theoretical infrared reflectance of the Cu–Bi film based on the measured dielectric constants in Supplementary Figure 13.

The above experimental results fill the missing link between the amorphous nanocluster nature and the enhanced broadband solar absorption and suppressed long-wave IR emissivity. To further address the alternative explanations raised by the reviewer, we clarify the following points:

**(1) Thickness effect.** We measured the solar absorption for films with different deposited thicknesses (controlled via charge density) and found that the absorption remains similarly strong with increasing thickness, indicating that enhanced absorption is insensitive to thickness (Supplementary Figure 17).

**(2) Surface roughness.** We characterized the morphology of these deposited materials via AFM (Supplementary Figure 18). Notably, the deposited Cu, Bi, and Cu–Bi exhibit similar surface roughness (Rq,_Cu_ < Rq,_Cu–Bi_ < Rq,_Bi_) which, therefore, cannot indicate the exotic optical behavior of Cu–Bi.

**(3) Percolation.** As discussed above, percolation primarily affects the electrical connectivity of the metallic network and is responsible for the suppressed mid-infrared emissivity, rather than the broadband solar absorption.

(4) Generic metal film**.** As demonstrated above, the comparison between Bi, Cu, and Cu–Bi clearly show that such broadband absorption is not a generic property of electrodeposited metals, but instead arises from the specific amorphous nanocluster structure of Cu–Bi.

Supplementary Figure 17. Absorptivity spectra of Cu–Bi films deposited on PE-graphene electrodes at varying charge densities (30, 60, 120, and 200 mC/cm²).

Supplementary Figure 18. AFM characterization of electrodeposited Cu (A), Bi (B), and Cu–Bi (C) surfaces. The root mean square roughness (Rq) and arithmetic mean roughness (Ra) are: Cu, Rq = 6.66 nm, Ra = 5.30 nm; Bi, Rq = 19.38 nm, Ra = 15.38 nm; Cu–Bi, Rq = 11.92 nm, Ra = 9.53 nm.

**Supplementary Discussion 4**

we have carried out a systematic study to correlate optical response, morphology, and crystallinity by independently varying the Cu:Bi ratio, deposition thickness, and pulsing parameters.

**1) Varying Cu:Bi ratio**

The Cu:Bi ratio was systematically tuned by varying the electrolyte composition while keeping the electrodeposition protocol fixed. Specifically, five electrolyte conditions were examined with varying Cu:Bi ratios (20:0, 15:5, 10:10, 5:15, and 0:20 mM). Supplementary Figure 19 shows that, in general, increasing Bi content in the Cu–Bi alloy increases the solar absorptivity while decreases the IR reflectivity. As a result, an intermediate Cu:Bi ratio (e.g., 10:10 mM) achieves a balanced optical response with simultaneously enhanced solar absorption and reduced infrared reflectivity. It is also noteworthy that, consistent with electrodeposition on Au-coated wafers (Supplementary Figure 12), co-deposition of Cu and Bi on PE/graphene yields stronger solar absorption compared to single-metal deposition (i.e., Cu-only or Bi-only).

Supplementary Figure 19. Effect of Cu:Bi ratio on optical response. (A) UV–Vis absorptivity and (B) mid-infrared reflectivity spectra of electrodeposited films with varying Cu:Bi ratios. All samples were prepared under identical nucleation pulse conditions (−3 V for 5 s), followed by a −1 V growth step with a total charge density of 120 mC cm⁻². All measurements were done using PE-graphene as the working electrode.

Supplementary Figure 20 shows the SEM characterization results of different samples. Consistent with the AFM characterization (Supplementary Figure 18), deposited Cu exhibits smaller particle size than Bi, and co-deposited samples show a mixed particle size distribution. The corresponding XRD results are shown in Supplementary Figure 21. While Bi-only samples display clear diffraction peaks characteristic of crystalline Bi, the introduction of a small Cu fraction (5Cu–15Bi) leads to a notable attenuation of these features. Upon increasing the Cu content to an equimolar ratio (10Cu–10Bi), diffraction peaks are nearly completely suppressed, indicating a transition to an amorphous structure. This amorphous character is retained in the Cu-rich composition (15Cu–5Bi), while the Cu-only sample recovers a well-defined crystalline diffraction peak.

Supplementary Figure 20. Effect of Cu:Bi ratio on surface morphologies characterized via SEM. Scale bar: 1 µm.

Supplementary Figure 21. Effect of Cu:Bi ratio on crystallinity characterized via XRD. The Bi-only sample (20Bi) shows distinct Bi diffraction peaks, which progressively attenuate with increasing Cu content. Near-complete peak suppression is observed at the equimolar ratio (10Cu–10Bi), indicating an amorphous structure. The Cu-only sample (20Cu) recovers a single crystalline Cu peak.

**2) Varying thickness**

The deposition thickness was systematically varied by adjusting the total charge density (30, 60, 120, and 200 mC cm⁻²) while keeping the electrolyte composition fixed at the 10Cu–10Bi ratio and the pulsing protocol unchanged (−3 V for 5 s followed by −1 V). As shown in Supplementary Figure 22(A), solar absorptivity initially increases with deposition thickness, reaching a maximum at an intermediate charge density, before declining at higher loadings. This non-monotonic behavior is attributed to competing effects: at moderate thicknesses, the rough nanoparticulate surface promotes multiple light scattering and optical path elongation, enhancing absorption; at excessive thicknesses, particle coalescence and increased surface coverage reduce inter-particle gaps, suppressing light trapping and allowing greater specular reflection from the denser metallic surface. Notably, solar absorptivity remains similarly high across all thickness conditions, underscoring the robustness of the Cu–Bi alloy film as a broadband solar absorber. In contrast, mid-infrared reflectivity increases monotonically with deposition thickness, consistent with the growing metallic character of thicker films.

Supplementary Figure 22. Effect of deposition thickness on optical response. (A) UV–Vis absorptivity and (B) mid-infrared reflectivity spectra of electrodeposited films with varying deposition charge density. All samples were prepared under identical pulsed deposition conditions (−3 V for 5 s), followed by a −1 V growth step with different charge densities. All measurements were done using PE-graphene as the working electrode.

SEM characterization (Supplementary Figure 23) reveals a clear morphological evolution with increasing charge density: the surface initially becomes denser with a higher particle number density, followed by progressive particle growth and coalescence at higher loadings. This trend is consistent with the optical observations and supports the interpretation that inter-particle scattering is most effective at intermediate thicknesses.

Supplementary Figure 23. Effect of deposition thickness on surface morphologies characterized via SEM. The samples are the same as those in Supplementary Figure 22. Scale bar: 1 µm.

XRD analysis confirms that all films, regardless of deposition thickness, remain amorphous, with no detectable diffraction peaks observed across the entire thickness series (Supplementary Figure 24). This indicates that the amorphous character of the 10Cu–10Bi alloy is an intrinsic property of the co-deposition chemistry rather than a thickness-dependent phenomenon.

Supplementary Figure 24. Effect of deposition thickness on crystallinity characterized via XRD. No diffraction peaks are observed across all charge densities (30–200 mC/cm²), confirming that the amorphous character of the 10Cu–10Bi alloy is preserved regardless of film thickness.

**3) Varying deposition parameter**

The effect of the nucleation pulse voltage was investigated by comparing two pulsed deposition conditions: −3 V and −2 V, both applied for a charge density equivalent to the standard nucleation step, followed by an identical −1 V growth step at 120 mC/cm². All depositions used the 10Cu–10Bi electrolyte composition.

As shown in Supplementary Figure 25, the −2 V sample exhibits higher solar absorptivity but lower mid-infrared reflectivity compared to the −3 V sample. SEM characterization (Supplementary Figure 26) suggests that the lower nucleation overpotential at −2 V generates a higher density of smaller nuclei, resulting in a more heterogeneous particle size distribution. This broader size distribution is expected to enhance broadband light trapping through multi-scale scattering, accounting for the improved solar absorption. However, the less densely packed, more irregular particle arrangement also compromises the percolation network of the metallic film, reducing its free-electron response and thus lowering mid-infrared reflectivity. These results demonstrate that the nucleation pulse voltage provides an additional handle for tuning the optical response of Cu–Bi alloy films by modifying surface morphology independently of composition and thickness.

Supplementary Figure 25. Effect of deposition parameter on optical response. (A) UV–Vis absorptivity and (B) mid-infrared reflectivity spectra of electrodeposited films with varying nucleation pulse voltage (−3 V for 5 s vs. −2 V at equivalent charge density), followed by an identical −1 V growth step at 120 mC cm⁻². All measurements were done using PE-graphene as the working electrode.

Supplementary Figure 26. Effect of deposition parameter on surface morphologies characterized via SEM. The samples are the same as those in Supplementary Figure 25. Scale bar: 1 µm.

Supplementary Figure 27 shows that both deposition conditions yield amorphous films, with no detectable diffraction peaks observed under either nucleation voltage, indicating that the crystalline character of the co-deposited alloy is independent of the nucleation pulse voltage.

Supplementary Figure 27. Effect of deposition parameter on crystallinity characterized via XRD. No diffraction peaks are observed under either deposition condition (−3 V or −2 V), confirming that the amorphous character of the 10Cu–10Bi alloy is preserved regardless of the nucleation pulse voltage.

We also conducted dedicated in-situ UV-Vis and in-situ XRD measurements during the electrodeposition process to correlate the structure and optical performance.

For the in-situ UV-Vis measurement, we designed and CNC-machined a custom electrochemical cell using an aluminum alloy body, with a PTFE spacer (2 mm thick) serving as the electrochemically inert chamber wall, and the working and counter electrodes mounted on either side of the spacer. A silver Mylar film was placed at the base to serve as a back reflector, and the assembled device was mounted directly onto a UV-Vis integrating sphere to enable real-time reflectance measurement throughout the full pulse-and-deposit sequence. We note that the 2 mm electrolyte path length introduces appreciable near-infrared absorption from the aqueous electrolyte; however, this background does not affect the qualitative optical trend observed during deposition. The absorptivity increases progressively with increasing deposition charge during the early stages, then decreases at higher deposition charges — a trend consistent with our previously reported ex-situ results.

To complement the optical measurements, we further performed in-situ XRD during electrodeposition using a thin-film compatible cell. In this configuration, PE films were used as X-ray transparent windows on both sides of the cell, replacing the silver Mylar used in the UV-Vis device so as to eliminate any confounding diffraction signal from the reflector. XRD profiles were collected continuously throughout the pulse-and-deposit sequence under the same electrochemical conditions. Critically, no crystalline diffraction peaks appeared at any stage of the deposition process, and the profiles remained featureless throughout, directly confirming that the Cu–Bi deposit remains amorphous during growth. This result rules out any transient crystalline phase formation during electrodeposition and provides direct in-situ evidence for the amorphous nature of the Cu–Bi nanoclusters.

Taken together, the in-situ UV-Vis and XRD results establish a clear and direct causal relationship: the electrodeposition process produces amorphous Cu–Bi nanoclusters in real time, and this structural evolution is accompanied by the characteristic enhancement in solar absorptivity observed both in-situ and ex-situ. We believe these new experiments comprehensively address the reviewer's concern and provide compelling in-situ evidence supporting the central conclusions of our work.

Supplementary Figure 28. In-situ UV-Vis absorptivity spectra recorded during Cu–Bi electrodeposition. A custom electrochemical cell mounted on a UV-Vis integrating sphere (right) was used to monitor absorptivity evolution at successive electrodeposition stages: pristine state, after an initial pulse step (−3 V), and at increasing deposition charges (30–180 mC/cm²) at −1 V. A 10Cu–10Bi electrolyte was used in the device.

Supplementary Figure 29. In-situ XRD profiles recorded during Cu–Bi electrodeposition. A thin-film electrochemical cell with PE film windows (right) was used to collect XRD profiles at successive electrodeposition stages: pristine state, after the initial pulse step (−3 V), and at increasing deposition charges (30–180 mC/cm²) at −1 V. A 10Cu–10Bi electrolyte was used in the device.

In summary, this systematic study deepens our mechanistic understanding of the Cu–Bi electrodeposition system, and demonstrates that solar absorption and infrared reflectivity can be flexibly tuned through rational control of the deposition conditions. More importantly, it highlights a distinctive feature of the Cu–Bi co-deposition system: an appropriate Cu:Bi ratio uniquely enables the formation of amorphous alloy films, a structural characteristic unattainable with single-metal deposition. This amorphous nature is remarkably robust across a wide range of deposition conditions, and is key to achieving the simultaneously enhanced solar absorption and infrared reflectivity that make these films promising for thermal management applications.

**Supplementary Discussion 5**

We have systematically evaluated the device stability under multiple real-world stressors, including UV exposure, thermal cycling, humidity, freeze-thaw cycling, and dust/soiling. The results are summarized below.

**1) UV Exposure**

Among all components of our device, the outer PE film electrode warrants the most concern regarding UV exposure, as polyethylene is known to degrade under prolonged sunlight due to its strong absorption of UV light. To assess its UV stability, we subjected the graphene-integrated PE film device to accelerated UV irradiation using a UV lamp (350 W/m²) at a 10 cm distance for 2 weeks. Since UV light constitutes approximately 4% of solar radiation, the natural UV intensity in summer is estimated at ~40 W/m² (assuming 1000 W/m² total solar intensity). Under this equivalence, 24 hours of lamp exposure corresponds to ~26.25 days of outdoor sunlight (assuming 8 daytime hours per day), meaning our 2-week irradiation protocol is equivalent to approximately one full year of outdoor UV exposure. After this treatment, UV-Vis and FTIR spectra were collected and showed little change compared to the pre-irradiation baseline, confirming excellent UV stability of the device.

Supplementary Figure 30. UV stability of PE/graphene films. (A) UV–Vis and (B) mid-IR transmittance spectra before and after 14 days of UV exposure.

**2) Thermal Cycling**

For thermal cycling tests, the device was assembled in a CNC-machined aluminum enclosure, where the upper and lower PE films were compressed and sealed by tightening screws to provide mechanical confinement. The assembled device was placed on a hot stage at 60 °C for 1 hour, then cooled to room temperature (20 °C) and held for 1 hour, constituting one thermal cycle; this was repeated for 10 cycles in total. The electrodeposition-induced mid-IR reflectance modulation was measured before and after cycling. As shown in the figure, heating to 60 °C caused partial softening of the PE film, which weakened the optical interference profile; however, the overall electrochromic tunability was preserved, demonstrating that thermal excursions to temperatures relevant to rooftop environments do not compromise device functionality.

Supplementary Figure 31**.** Thermal cycling stability of the device. Mid-IR reflectivity spectra before and after 10 thermal cycles (between 60 °C and 20 °C) in both ON and OFF states.

**3) Humidity**

To evaluate moisture robustness, the device was placed in a sealed enclosure containing an ultrasonic humidifier and maintained at an average relative humidity of over 95% for 24 hours. After removal and re-equilibration to ambient conditions, the IR tunability was measured and found to be essentially unchanged compared to the pre-exposure baseline. This demonstrates that the device is robust against high-humidity environments encountered in outdoor deployment.

Supplementary Figure 32**.** Humidity stability of the device. Mid-IR reflectivity spectra before and after high-humidity exposure (>95% RH, 24 h) in both ON and OFF states.

**4) Freeze-Thaw Cycling**

The device was placed on a temperature-controlled stage set to −20 °C for 4 hours to ensure complete freezing of the electrolyte, followed by thawing to room temperature. Post-thaw FTIR measurements showed that the electrochromic tunability was well preserved relative to the pre-freeze state, confirming that freeze-thaw events do not cause irreversible degradation to device performance.

Supplementary Figure 33**.** Freeze–thaw stability of the device. Mid-IR reflectivity spectra before and after freeze–thaw treatment (−20 °C to room temperature) in both ON and OFF states.

**5) Dust/Soiling**

Since only the outer PE film is exposed to ambient particulates in a practical rooftop installation, we performed soiling tests on bare PE film samples. Soil collected from an outdoor site was dispersed in deionized water and uniformly applied to the PE film surface; the coated samples were allowed to air dry completely, after which UV-Vis and FTIR transmittance were measured. The soiled film exhibited a slight reduction in transmittance, consistent with light scattering by adsorbed particulates (the magnitude of which scales with dust loading). The film was then gently rinsed with deionized water and air dried, and the transmittance was remeasured. Transmittance recovered to near the original value, indicating that particulate contamination is readily removed by light rainfall. This self-cleaning behavior is attributed to the intrinsically low surface energy and hydrophobic character of PE, which inhibit strong adhesion of dust particles and facilitate their removal by water. Therefore, our device design is robust against dust and soiling under realistic outdoor conditions.

Supplementary Figure 34**.** Dust/soiling robustness of PE films. (A) UV–Vis and (B) mid-IR transmittance spectra of pristine, contaminated, and post-cleaned samples.
